# The HOCl dry fog–is it safe for human cells?

**DOI:** 10.1371/journal.pone.0304602

**Published:** 2024-05-29

**Authors:** Rafał Bogdan Lewandowski, Małgorzata Stępińska, Łukasz Osuchowski, Wiktoria Kasprzycka, Monika Dobrzyńska, Zygmunt Mierczyk, Elżbieta Anna Trafny

**Affiliations:** Institute of Optoelectronics, Biomedical Engineering Centre, Military University of Technology, Warsaw, Poland; Universidade Federal do Para, BRAZIL

## Abstract

This study aims to investigate if high-concentration HOCl fogging disinfection causes cytotoxicity and genotoxicity to cultured primary human skin fibroblasts. The cells were exposed to a dry fog of HOCl produced from solutions with a concentration of 300 ppm (5.72 mM) or 500 ppm (9.53 mM). After four times when fibroblasts were exposed to aerosolized HOCl at a concentration of 500 ppm for 9 minutes, significant cytotoxicity and genotoxicity effects were observed. Significant changes in the morphology of fibroblasts and cell death due to membrane disruption were observed, independent of the number of exposures. Flow cytometry analyses performed under these experimental conditions indicated a decrease in the number of cells with an intact cell membrane in the exposed samples compared to the sham samples, dropping to 49.1% of the total cells. Additionally, under the same conditions, the neutral comet assay results demonstrated significant DNA damage in the exposed cells. However, no analogous damages were found when the cells were exposed to aerosolized HOCl generated from a 300-ppm solution for 3 minutes, whether once or four times. Therefore, we have concluded that aerosolized HOCl in dry fog, with a concentration exceeding 300 ppm, can cause cytotoxic and genotoxic effects on human skin fibroblasts.

## Introduction

Aerosol hypochlorite solutions were proposed as an effective measure against airborne pathogens in London hospitals as early as the 1940s [1]. Whenever a disinfectant is used as an aerosol [2], e.g., fog or mist, the question always arises as to the safety of its use in the presence of humans [3]. Most previous studies on the cytotoxicity of hypochlorous acid have been performed on cell lines treated with a hypochlorous acid solution [4–6]. No adverse effects of hypochlorous acid were observed at concentrations ranging from 100 ppm [2] to 1000 ppm and even up to 1300 ppm. More literature is available on the effects of sodium salt and hypochlorous acid and its effects on various organisms, including bacteria, fungi, and viruses [7]. According to Yan et al. [8] and da Cruz Nizer et al. [9], hypochlorous acid has 80 times higher bactericidal activity than OCl¯ due to its ability to migrate through the lipid membrane bilayer [10]. The potential antimicrobial mechanisms of HOCl acid have been described in detail in previous studies [8,9]. The denaturation of proteins and their subsequent aggregation within cell membranes, especially those involved in energy transfer and transport within a cell, are considered the primary mechanisms of HOCl’s deleterious effects. This results in inhibition of ATP production and disruption of proper transport chains in physiological cell processes [8,9].

Few studies have evaluated the cytotoxicity of aerosolized hypochlorous acid on human cells cultured *in vitro* [11,12]. It should be the first step in conducting research on the potential mechanisms of disruption of human cell homeostasis due to exposure to aerosolized hypochlorous acid [13]. The claims of non-toxicity of this disinfectant are based on animal studies [11,14] and exposure of volunteers [15]. While the authors of these articles state that no adverse effects occurred when mammals were exposed to aerosolized hypochlorous acid, we could not find specific experimental results to support these claims. In addition, adverse effects of HOCl solution on erythrocytes [16], human umbilical vein endothelial cells (HUVEC) [17], ovarian carcinoma tumor cells (SK-OV-3) [18], and the human epithelial lung adenocarcinoma cell line (A549) [19] have been reported. The following mechanisms have been investigated: cell membrane damage [20], apoptosis [17], necrosis [18], DNA degradation/genotoxicity [17,19] and mutagenicity [19]. It has been shown that cell damage occurs via the above mechanisms when HOCl concentrations exceed physiological levels in tissues. However, none of the aforementioned papers consider the effect of hypochlorous acid acting as a dry fog on cells.

The Biocidal Products Committee (BPC) of the European Chemicals Agency (ECHA) has approved aqueous solutions of HOCl releasing 200–300 ppm of active chlorine for hard surface disinfection in the EU [21]. Therefore, a pertinent question is whether it is feasible to disinfect surfaces in the presence of the operator using hypochlorous acid at concentrations of 300 ppm or 500 ppm. To address this question, we performed experiments in which human primary fibroblasts were exposed to HOCl aerosol. Our studies used primary (non-immortalized) human fibroblasts, which may be more sensitive to dry fog HOCl exposure. Fibroblasts are the primary cells of connective tissue and are part of the skin, which is the primary protective barrier of the human body against infectious pathogens. They synthesize components of the extracellular matrix of tissues, including collagen, and actively participate in tissue healing processes. Fibroblasts can, therefore, be treated as cells whose normal functions indicate the body’s homeostasis after exposure to potentially harmful chemical or physical factors. In addition, primary lines are characterized by high biological relevance and maintain genetic integrity at levels similar to *in vivo* conditions [22]. Despite their limited lifespan, they are good experimental models for *in vivo* studies. We evaluated cell morphology, viability, apoptosis, and DNA damage after exposure to HOCl dry fog or water aerosol.

## Materials and methods

### Human fibroblasts

Primary human skin fibroblasts (LONZA, CC-2511 NHDF) were cultured in the FGM-2 Fibroblast Bullet Kit medium with FBMTM Basal Medium (CC-3131, Lonza), FGMTM-2 SingleQuotsTM supplements (CC-4126, Lonza), and a mixture of antibiotics (Pen/Strep/Fungizone, 17-745E, Lonza) in a tissue culture incubator (INCOmed 153, Memmert) in an atmosphere of 5% CO_2_ at 37°C and 90% humidity. For experiments, cultured cells were thawed between passages 4 and 7, and cell growth was monitored under an inverted microscope (Nikon TS2RFL). The cell density at seeding was approximately 4 x 10^4^ cells/well on the 6-well culture plate. The volume of medium used in each well was 2.0 mL and was mentioned the next day.

### Hypochlorous acid aerosol

HOCl solutions at concentrations of 300 ppm (5.72 mM) (BMAsept surf&air) and 500 ppm (9.53 mM) (BMAsept air) were provided by BioMedAqua, Dębica, Poland. Aerosol was generated using a pneumatic generator (nozzle) equipped with a one-way valve that automatically shuts off the air source. The concentration of aerosolized particles was constantly monitored every 30 seconds with the Abakus^®^ Mobil Air Particle Counter for Air and Gases (Klotz GmbH) in a flow of 2.8 dm^3^ per min (±5%) during all experiments. The volume of air drawn into the system was 1.4 dm^3^. The total number of particles was measured in the sampled volume of air. The volume of the aerosolized HOCl solution was 108 mL. The volume of the test chamber was equal to 5.88 m^3^. As a result, the total concentration of HOCl introduced into the chamber volume was 0.618 mM in the case of experiments using a 300 ppm (5.719 mM) solution, whereas, in experiments using a 500 ppm (9.531 mM) solution, the HOCl concentration in the chamber was 1.029 mM. Once the human cells were exposed, the chamber was sealed, and the ventilation system started purging the chamber before the next fogging event. The supplemental air was supplied to the chamber through the HEPA filter. The time interval between subsequent aerosol generation cycles was set at 40 minutes and remained consistent for both HOCl concentrations of 300 ppm and 500 ppm.

### Human cells’ exposure to hypochlorous acid

For the experiments, 6-well culture dishes were inoculated with the fibroblast suspension at the density 4 x 10^4^ cells/well and were cultured for 3 days before start of HOCl fogging experiment. S1 Fig shows the morphology of fibroblasts grown for three days in control samples. Three types of samples were investigated. Control samples contained fibroblasts that had not undergone any treatment and were continuously stored in an incubator under optimal growth conditions. Sham samples contained fibroblasts that were not exposed to dry HOCl mist and were kept in an additional chamber close to the HOCl exposure chamber for the duration of the exposure. They were protected from HOCl exposure but experienced the same environmental conditions (temperature, humidity) as the exposed samples. Exposed samples contained fibroblasts directly exposed to dry HOCl fog in the HOCl exposure chamber under the same environmental conditions (temperature, humidity) as the sham samples. After aspirating the culture medium, the cells were exposed to HOCl aerosol at room temperature. The plates were placed vertically in the chamber to prevent droplets from settling on the surface of the adherent cells. After exposure, the cell suspensions from four consecutive wells were combined and processed as one exposed sample (one biological replicate). Experiments were performed with three biological replicates per condition. At the same time, sham samples underwent the same processing. For this purpose, sham samples without medium were exposed to a sterile ultrapure H_2_O aerosol for 2 minutes using a humidifier Humidifier hydro SPA (Esperaza). Fibroblasts from both sham and control samples were combined similarly to the exposed cells and then processed in the three biological replicates. The number of fibroblasts obtained for each sample after the cell detachment (i.e., biological replicate) ranged from 5 × 10^5^ to a maximum of 5 × 10^6^ cells/mL for individual experiments.

Fibroblasts were exposed to HOCl or water aerosol one or four times. After each exposure (cycles 1–4), 2 mL of culture medium was added to each well and incubated for 30 minutes in a CO_2_ incubator at optimal temperature. Before the next fogging cycle, the culture medium was removed, and adherent cells were exposed to aerosolized HOCl or H_2_O. After each cycle, the removed culture medium was collected and kept in the culture incubator, and the detached cells were added to the total cell pool (a biological replicate). After the last fogging cycle, the adherent cells were detached using 0.025% (w/v) trypsin solution (Lonza CC-5012) from the Reagent Pack Subculture Kit (Lonza CC-5034). Prior to trypsinization, the cell layer was washed with HEPES buffer, and trypsin/EDTA solution was added (1 mL per well of a 6-well plate). After centrifugation at 225 × g for 5 minutes, cells from four wells were suspended in 900 μL of medium (a biological replicate). The density and viability of cell suspensions were measured using a Countess^TM^ cell counter (Life Technologies). Fibroblasts were observed for distinct morphology in an inverted diagnostic microscope (Nikon TS2RFL) between successive exposures. Photographs were taken at 4 × and 10 × magnification using a microscope-integrated camera and specialized software. Each experiment was repeated twice. Fibroblasts were monitored for distinctive morphology in an inverted diagnostic microscope (Nikon TS2RFL) between successive exposures. Photos at magnifications of 4 and 10 times were captured using a camera integrated with a microscope and specialized software. Each experiment was repeated two times.

### Fibroblast viability under scanning confocal microscope

The Invitrogen™ LIVE/DEAD™ Viability/Cytotoxicity Kit for mammalian cells was used according to the manufacturer’s instructions. Cells were observed immediately after exposure to HOCl aerosol. Intracellular esterase activity caused calcein AM in live cells to produce green fluorescence upon excitation. Conversely, ethidium homodimer-1 (EthD-1), a red fluorescent dye, binds to nucleic acids in cells whose plasma membranes have been damaged. The starting solution had a concentration of 2 μM calcein AM and 4 μM EthD-1. Immediately after exposure, all cell samples were flooded with a warm (37°C) solution of fluorescent dyes in PBS. Experiments were performed in at least two replicates per sample. Cell morphology and viability were observed after incubation in the dark at 37°C for 15 minutes using a confocal fluorescence microscope (LSM 700 Axio Observer.Z1 Zeiss). Images of whole wells and cell groups from samples with damaged cells were captured.

### Fibroblast viability by flow cytometry

Fibroblasts at a density of 2–5 × 10^5^ cells/mL were centrifuged, washed twice with PBS, and resuspended in cytometry buffer (Pharm Stain Buffer, Becton Dickinson 554656). Cells were filtered through sieves (Becton Dickinson) to exclude cell clusters. The resulting cell pellets were resuspended in 100 μL of cytometry buffer and stained with the following fluorescent dyes: Alexa Fluor 488 conjugated to annexin V (Annexin V-FITC Apoptosis Detection Kit II, 556570, Becton Dickinson) (5 μL) and propidium iodide (PI) (from Tali™ Apoptosis Kit—Annexin V Alexa Fluor™ 488 and Propidium Iodide, Invitrogen) (5 μL) for 15 minutes at room temperature. Sham samples were used as negative controls. Samples were analyzed on a FACSAria™ III flow cytometer using BD FACSDiva software version 10.0 (New Jersey, USA) with a minimum of 10^4^ cells per sample. The analysis resulted in the calculation of the cell population in four categories: live (Annexin V-negative, PI-negative); apoptotic (Annexin V-positive, PI-negative); late apoptotic/necrotic (Annexin V-positive, PI-positive); and dead (Annexin V-negative, PI-positive). Gates were set in the green channel 530/30 for Alexa Fluor 488 and in the yellow channel 585/45 for PI. An argon laser (488 nm) was used for excitation. Analyses were performed 1.5 hours after fibroblast exposure.

### Comet assay

The OxiSelect™ Comet Assay Kit (Cell Biolabs, Inc., STA-355, San Diego, CA) was used according to the manufacturer’s instructions. The steps of comet assay (conditions and results) were described taking into account essential requirements presented in Consensus Statement for the Minimum Information for Reporting Comet Assay (MIRCA) [23]. Immediately after exposure, the cell suspension was adjusted to a density of 1 × 10^5^ cells per mL in ice-cold PBS (without Mg^2+^ and Ca^2+^) and then resuspended in OxiSelect™ Comet Agarose solution at a final concentration of 10% (v/v) at 37°C. The OxiSelect™ Comet Slide was then loaded with 75 μL/well cell/agarose mixture, and the slides were transferred to 4°C in the dark for 15 minutes. The comet slides were then incubated in a cold fresh lysis buffer (2.5 M NaCl, 100 mM Na_2_ EDTA, 10 mM Tris, 1% Triton X-100, 10% DMSO, pH 10) in the dark for 60 minutes at 4°C. The buffer was then replaced with pre-chilled alkaline buffer (300 mM NaOH, 1mM Na_2_ EDTA, pH 13) and kept at 4°C for 30 minutes. The samples were then transferred to fresh, cold TBE electrophoresis solution (Tris-borate-EDTA buffer) for 5 minutes, and the process was repeated. Finally, the slides were placed on a horizontal gel electrophoresis unit filled with TBE electrophoresis solution. Electrophoresis was performed at 1.3 V/cm for 20 minutes at room temperature. The samples were then rinsed three times with distilled water, immersed in cold 70% ethanol for 5 minutes, and dried. The slides were stained with ethidium bromide (20 μg/mL) for 15 minutes at room temperature. Cells were observed using a Nikon Eclipse epifluorescence microscope with 20× objective magnification. At least 70 cells per slide were imaged using a digital imaging system for each experiment. To avoid additional DNA damage, all of the above procedures were performed under dim light. A Nikon DSFi1-U2 camera equipped with Nikon NISF soft-ware was used to capture images. Fibroblasts treated with 50 μM etoposide (Sigma-Aldrich, St. Louis, MO) for 24 hours were used as a positive control to detect DNA damage in human fibroblasts. Fibroblasts incubated at optimal growth conditions were used as a negative control.

### Comet data analyses

CaspLab^®^ software (2020, http://casplab.com) was used to analyze DNA damage. To determine the extent of DNA damage, the percentage of DNA in the comet tail was quantified, and the tail moment was calculated as the percentage of DNA in the tail multiplied by the length of the tail. In addition, according to Focke et al. [24], the nuclei were classified into five categories according to the DNA content in the tail: the cells with less than 5% DNA in the tail were classified as stage A, while cells with 5–20% DNA in the tail were classified as stage B. Similarly, cells with 20–40% DNA and 40–95% DNA in the tail were classified as stage C and D, respectively. Finally, cells with more than 95% DNA in the tail were classified as stage E. Tail DNA% and tail DNA moment data were expressed as the median of four independent experiments, with at least 280 comets scored for the positive control, sham, and exposed groups. DNA damage in fibroblast was demonstrated as boxplots were each box shows the median value (the horizontal line within a box) of three biological replicates per condition. The 25th and 75th percentiles are indicated by the bottom and top of the boxes, respectively. Whiskers: 5th and 95th percentiles. The non-parametric Mann-Whitney U test (Statistica v. 13.3.721.1 for Windows) was used to evaluate the significant difference between the exposed and sham groups. The percentages of cells in the comet stage categories were expressed as the mean ± standard deviation (SD) of three biological replicates per condition. The significant difference between the exposed and sham groups was determined and p-values were calculated using paired Student’s t-test. The statistical significance level was set at p<0.05.

## Results

### HOCl aerosol particles size distribution

**Fig 1:**
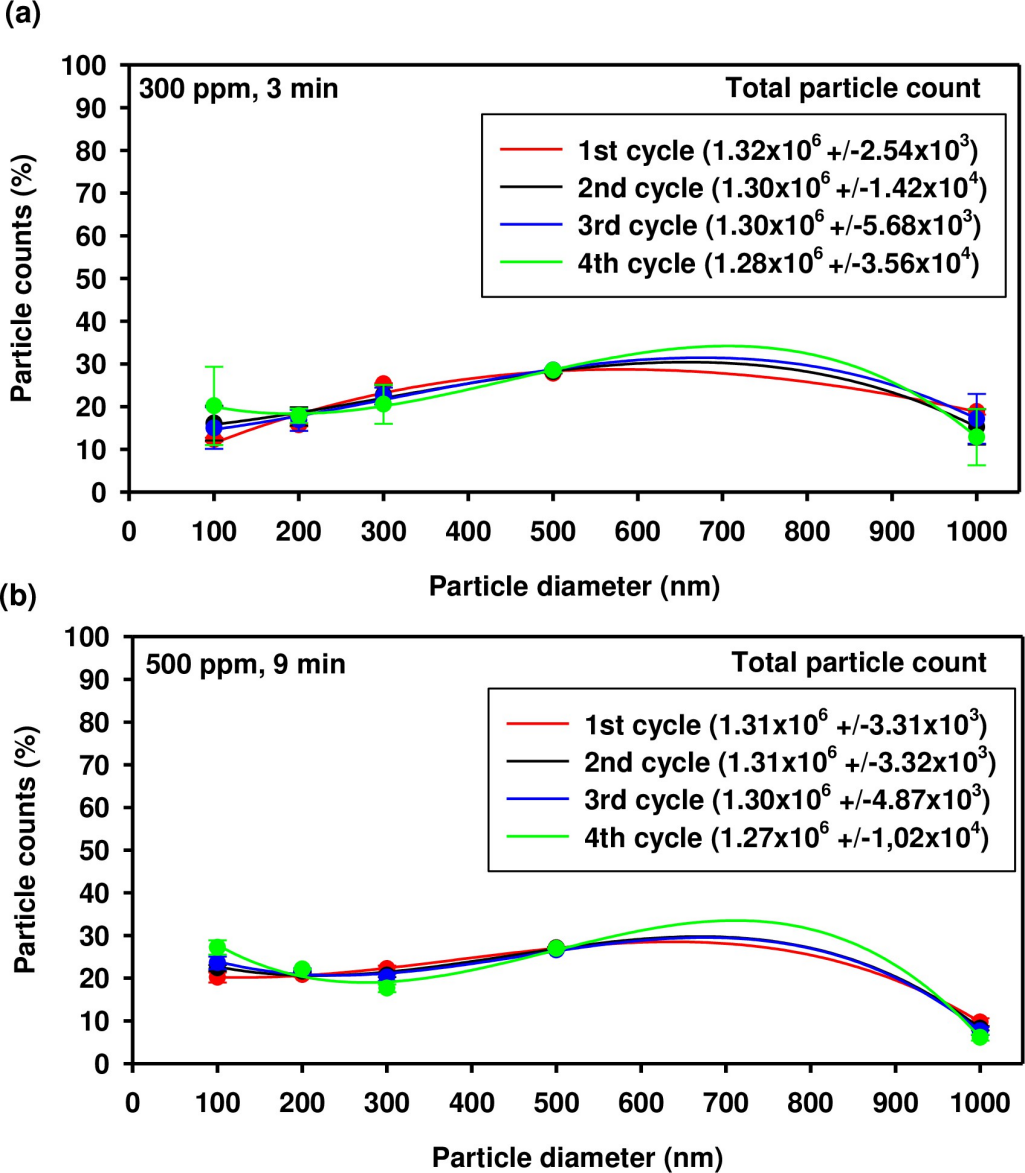
The distribution of size and number of particles of the generated HOCl aerosol at a concentration of 300 ppm (a) or 500 ppm (b) estimated for subsequent fogging cycles. The total number of particles was measured in the sampled air volume. Each data point represents the mean and standard deviation of four independent measurements.

The hypochlorous acid aerosol monitoring results indicate that the generated fog was homogeneous and reproducible. The total number of particles in the aerosol remained consistent throughout. Similar size distributions were observed for aerosol particles generated from 300 ppm and 500 ppm solutions as shown in Fig 1A and 1B. Most of these particles were smaller than 10 μm in diameter, i.e. that the HOCl was produced as so-called dry fog. The total number of aerosol particles was similar and exceeded 10^9^ particles in a volume equal to 5.88 m^3^. The free chlorine concentration did not exceed the EU short-term occupational exposure limit (0.5 ppm) during the cell exposure time.

### Fibroblast morphology after exposure to HOCl aerosol

**Fig 2:**
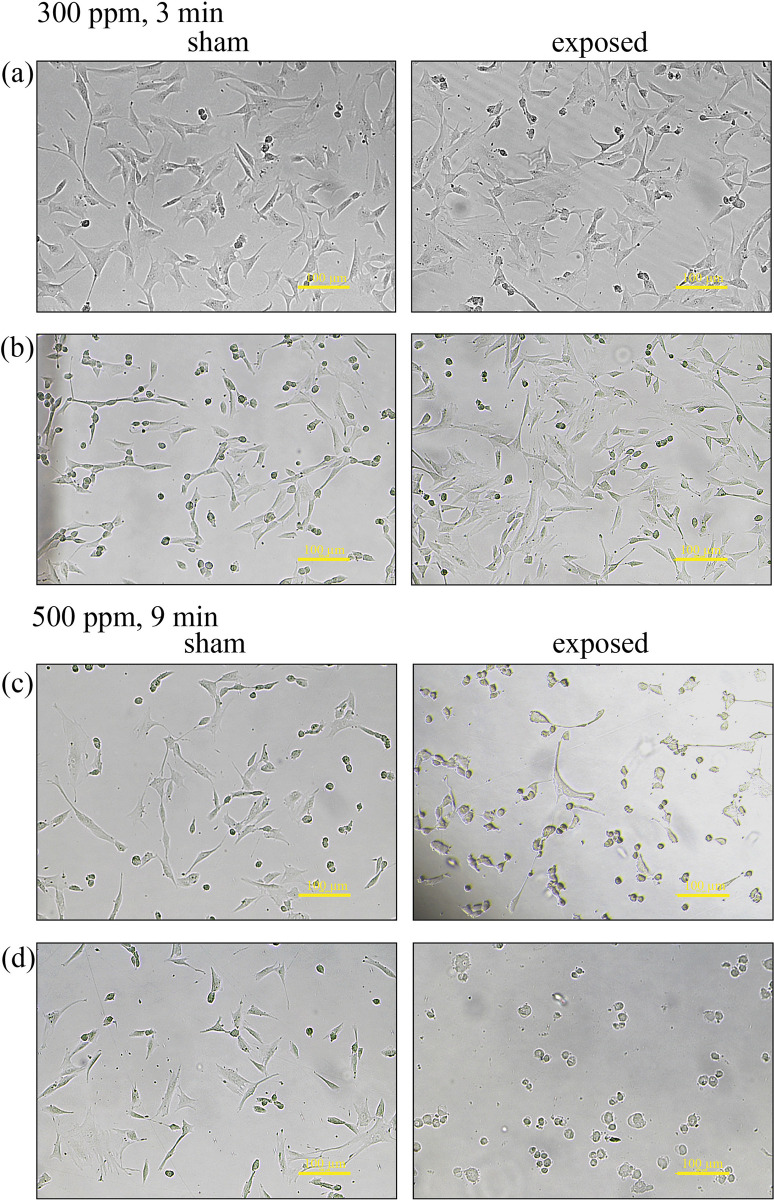
The cell morphology of adherent human fibroblasts under an inverted diagnostic microscope. Cells were exposed to 300 ppm HOCl dry fog for three minutes, either during a single cycle (a) or for four cycles (b), or to 500 ppm HOCl dry fog for nine minutes, either during a single cycle (c) or for four cycles (d). A 100-μm-scale bar is included.

Healthy human fibroblasts growing adherently to the bottom of the culture vessel exhibited characteristic elongated, spindle-shaped, and irregular shapes. Upon treatment with HOCl aerosol, two distinct morphological changes occurred depending on the oxidant concentration. After exposure to 300 ppm HOCl for 3 minutes for one cycle (C1) and four cycles (C4), no significant changes in cell morphology were observed (Fig 2A and 2B). Exposure of sham samples to water aerosol in a humidity chamber served as an effective negative control in our experiments, and no significant changes in cell morphology were also observed in sham samples after such treatment. Both sham- and HOCl-exposed cells showed only single round cells among the healthy spindle-shaped, elongated fibroblasts.

In contrast, significant changes in cell morphology were noticed after exposure to 500 ppm HOCl for 9 minutes following cycles C1 and C4 (Fig 2C and 2D). Differences between the sham and exposed samples were noted after one cycle of exposure to HOCl at a dose of 500 ppm (Fig 2C). Exposed samples exhibited an increased number of cells with an altered rounded morphology. After the fourth aerosolization cycle, fibroblasts with normal spindle-shaped morphology were no longer observed. The culture was destroyed, and the remaining fibroblasts at the bottom of the culture vessel rounded up and became poorly visible under the microscope (Fig 2D).

The above observations confirm that an aerosol of HOCl, with a concentration of 300 ppm, did not affect fibroblasts in adherent *in vitro* culture after 3 minutes of exposure. After exposure to the afore mentioned dose of HOCl, human skin-derived fibroblasts still exhibited the characteristic spindle-shaped shape of cells in adherent culture. Increasing the exposure dose of HOCl from 300 ppm to 500 ppm and extending the aerosolization time to 9 minutes resulted in negative effects on fibroblast culture. Several cell morphological changes were observed, including alterations in cell shape and detachment of cells from the culture vessel’s bottom.

### Fibroblast viability under scanning confocal microscope

**Fig 3:**
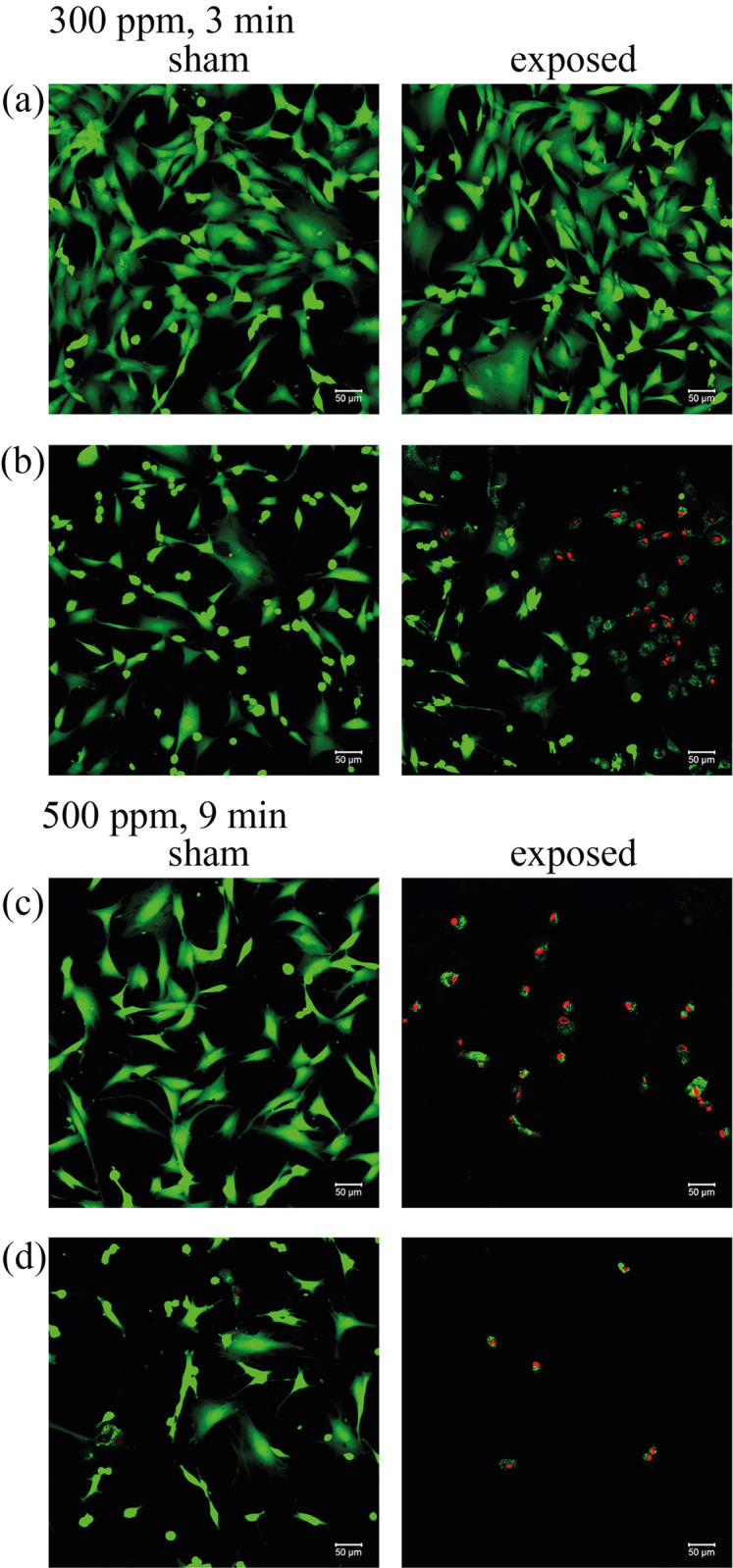
Morphology of fibroblasts under a scanning confocal microscope after exposure to HOCl aerosol. Cells were exposed to HOCl aerosol at a concentration of 300 ppm for three minutes after one (a) and four (b) exposure cycles or at a concentration of 500 ppm for nine minutes after one (c) and four (d) exposure cycles. The condition of the cell membrane was assessed by fluorescence staining; green fluorescence indicates no damage, and red indicates membrane damage. The white bar represents 50 μm.

Microscopic images of fibroblasts taken after exposure to HOCl aerosol allow visualization of membrane damage and intracellular esterase activity, thus assessing cell viability. The kit facilitated the observation of cell membrane disruption, indicated by ethidium homodimer-1 penetrating the cell and staining the nucleus red. Cells containing active intracellular esterases hydrolyzed the acetoxymethyl ester, converting the added dye into green fluorescent calcein. An intermediate stage consisting of green-stained cells was also observed, indicating severe metabolic disorders that inhibit the action of esterases. No red staining of the nucleus or loss of plasma membrane integrity was observed. In addition, these microscopic observations allowed the evaluation of changes in cell shape between sham and exposed cells. Cells exposed to HOCl aerosol at a concentration of 300 ppm for 3 minutes showed no changes compared to the sham after a single exposure (Fig 3A and 3B). However, after quadruple exposure, a reduction in cell flattening was observed, along with cells with disturbed metabolism (patched) and broken cell membranes. Exposure of cells to a 500-ppm concentration of HOCl aerosol for 9 minutes resulted in cell death visible by disruption of their membrane continuity. This phenomenon was observed regardless of the number of HOCl exposures. At four exposures, detachment of cells from the surface was documented (Fig 3C and 3D).

### Fibroblast viability by flow cytometry

**Fig 4:**
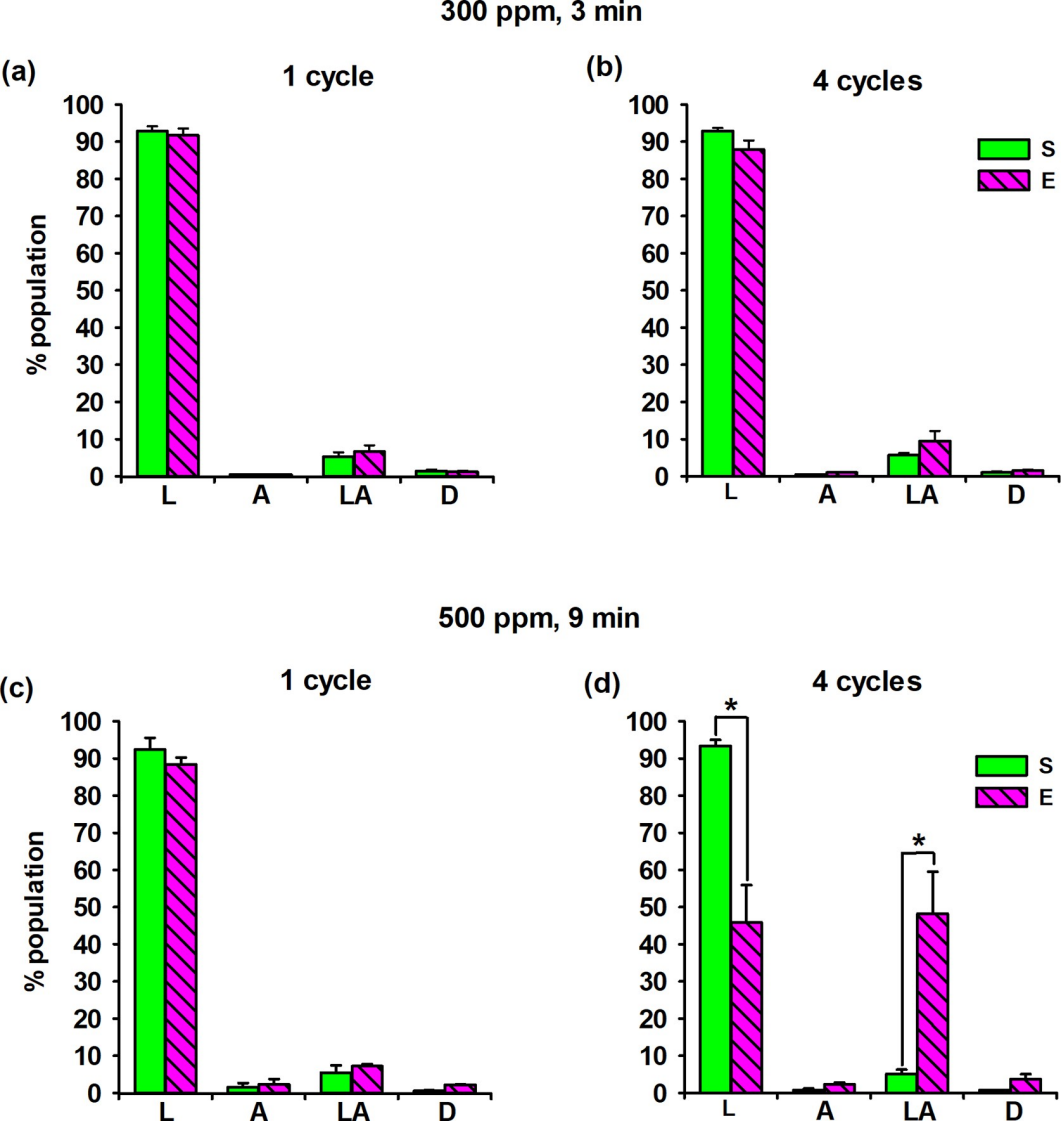
Flow cytometric apoptosis detection by Annexin V-FITC and PI double staining after HOCl fogging. S, sham-exposed cells. E, HOCl fog exposed cells. Four fibroblast populations were identified: L, viable; A, early apoptotic; LA, late apoptotic; D, necrotic. They were distinguished by flow cytometry, and 10.000 events were recorded for each sample. The percentage of cells in the following populations was evaluated. The bars represent the mean values (± SD) with three biological replicates per condition; differences were significant (Student’s t-test (* p<0.05)).

Exposure to HOCl aerosol at a concentration of 300 ppm for 3 minutes, either once or four times, did not change the proportions of cells with intact or damaged cell membranes compared to sham-exposed samples. The percentage of cells in apoptosis and late apoptosis was consistent in both experiments, indicating that the cell membrane was not damaged beyond physiological levels under *in vitro* conditions (Fig 4A and 4B). However, exposure to 500 ppm HOCl aerosol for 9 minutes increased the number of cells with damaged cell membranes. After the first exposure, 95.6% of the cells in the exposed samples had intact membranes (were alive) (Fig 4C). However, after four exposure cycles, the number of cells with intact membranes decreased to 49.1% compared to the sham samples (Fig 4D). These data indicated a significant decrease in fibroblast viability in the exposed samples compared to the sham samples. In addition, the proportion of apoptotic and post-apoptotic cells significantly increased, indicating cell membrane damage and the occurrence of cell death by apoptosis in the HOCl-treated cells.

### Damage of DNA in fibroblasts

**Fig 5:**
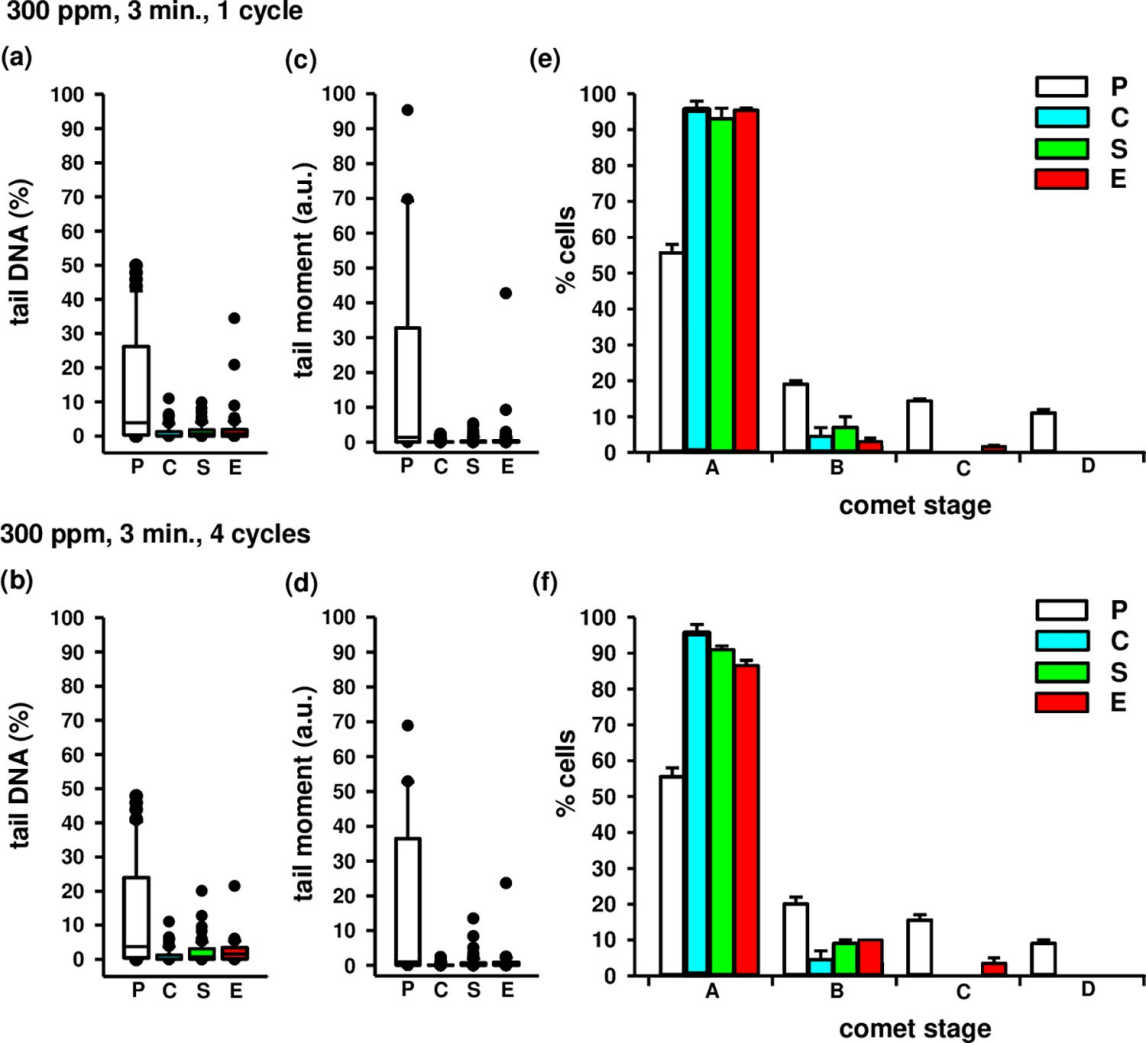
DNA damage in fibroblasts exposed to HOCl at a concentration 300 ppm. S, sham-exposed cells. E, exposed cells. The extent of DNA damage was evaluated 30 minutes after HOCl exposure by the neutral comet assay and expressed as tail DNA ((a)–one cycle, (b)–four cycles) or tail moment ((c)–one cycle, (d)–four cycles). C, unexposed cells, a negative control. P, the cells treated with 50 μM etoposide for 24 h, positive control. The percentages of cells in comet stage categories ((e)–one cycle, (f)–four cycles).

The neutral comet assay quantified DNA damage (double-strand breaks) within 30 minutes of cell exposure to HOCl aerosol. Human fibroblasts did not exhibit significant DNA damage after 300 ppm HOCl aerosol exposure for 3 minutes. However, the median percentage of damaged DNA in the comet tail of the exposed samples was higher than that in the comet tails of the sham samples after one or four exposures to HOCl aerosol. These values were 0.78 and 1.53% for the exposed samples and 0.63 and 0.76% for the sham samples, respectively. However, these differences were not significant in either experiment (p>0.05) (Fig 5A and 5B).

Meanwhile, the median percentage of tail DNA for the positive control (etoposide-treated cells) was 3.89 and 3.74%, respectively (Fig 5A and 5B). There was no significant difference (p>0.05) between the mean tail DNA percentages of exposed versus sham-treated cells. The respective values were 0.17, 0.23, 0.04, and 0.07 arbitrary units (a.u.). Median tail moments for positive controls were 1.36 and 1.01 a.u. (Fig 5C and 5D). In addition, this study assessed the nucleus DNA damage according to a classification that distinguishes five comet stages. Stage A denoted cells without nuclear DNA damage, while stages B, C, and D denoted low, intermediate, and high nuclear DNA damage. No comets in stage E representing cells with complete nuclear DNA damage were observed in this study. After 3 minutes exposure to the oxidant at a concentration of 300 ppm, the percentage of fibroblasts in comet stages B, C, or D showed no significant differences compared to the corresponding sham-exposed fibroblasts (Fig 5E and 5F). Exposed samples showed a similar pattern to the corresponding sham-exposed cells, whereas the distribution pattern of comet stages B-D varied between positive control (etoposide) and exposed samples (Fig 5E and 5F).

**Fig 6:**
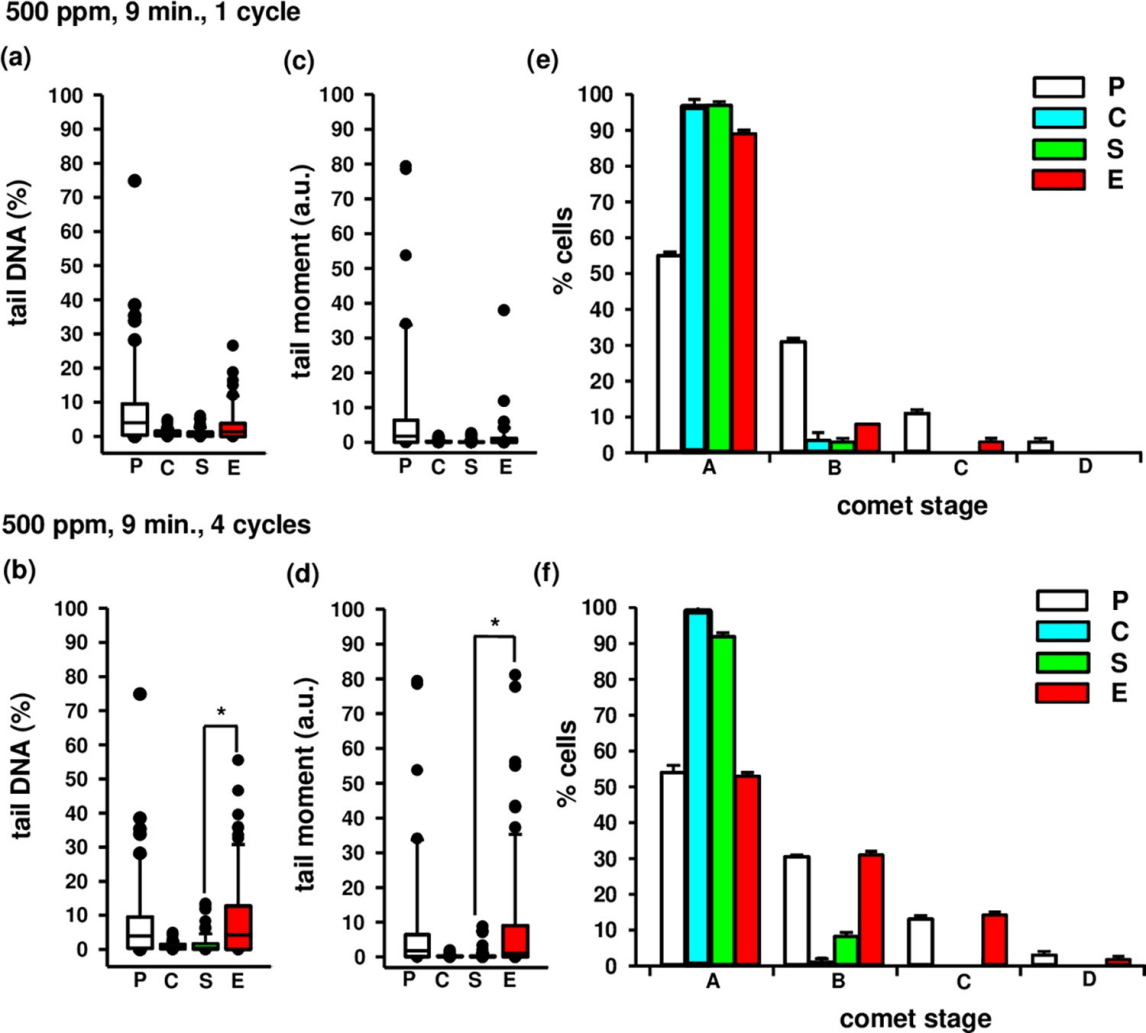
Neutral comet assay results showing DNA damage in fibroblasts exposed to HOCl at a concentration 500 ppm. S, sham-exposed cells. E, exposed cells. The extent of DNA damage was evaluated 30 minutes after exposure by the neutral comet assay and expressed as tail DNA ((a)–one cycle, (b)–four cycles) or tail moment ((c)–one cycle, (d)–four cycles). C, unexposed cells, a negative control. P, the cells treated with 50 μM etoposide for 24 h, positive control. A significant difference between sham- and HOCl-exposed cells was determined with the nonparametric Mann–Whitney U test (* p<0.001). The percentages of cells in comet stage categories ((e)–one cycle, (f)–four cycles).

Exposure to a one-time dose of 500 ppm of HOCl aerosol for 9 minutes did not induce significant DNA damage in human fibroblasts, as shown in Fig 6A, 6C and 6E.

Although the median percentage of damaged DNA in the comet tail was higher in exposed samples than in sham samples at 1.29 and 0.67%, respectively, there were no significant differences in either sample (p>0.05) (Fig 6A). Meanwhile, the median percentage of tail DNA for the positive control (etoposide-treated cells) was 4.29% (Fig 6A). In addition, no significant differences (p>0.05) were observed in the median tail moment values in the exposed samples compared to the corresponding sham samples. These values were 0.21 and 0.04 a.u., respectively (Fig 6C). The median tail moments for the positive controls were 1.70 and 1.01 a.u., respectively (Fig 6C). Between the positive control and the exposed samples, the distribution pattern of comet stages B to D was similar. However, the corresponding distribution for exposed samples was no significantly different from sham-exposed cells (Fig 6E).

However, exposure to HOCl aerosol at a concentration of 500 ppm four consecutive times for 9 minutes resulted in significant DNA damage in human fibroblasts (Fig 6B, 6D and 6F). In the exposed samples, the median percentage of damaged DNA in the comet tail was significantly higher than in the sham samples at 4.25% and 0.41%, respectively (p<0.05) (Fig 6B). At the same time, the median percentage of tail DNA for the positive control was 4.29% (Fig 6B). In addition, the median tail moments of 1.02 and 0.02 a.u. for exposed and sham samples were significantly different in these experiments (p<0.05). The median tail moment of the positive control was also 1.67 a.u. (Fig 6D). As shown in Fig 6F, the distribution pattern of comet stages B to D in the positive control and exposed samples was similar. This suggests that the mechanism of DNA damage in the exposed samples is similar to that in the positive control. Etoposide, as a topoisomerase II inhibitor, induces double-strand breaks in cells. Furthermore, the distribution of exposed samples did not resemble that of the corresponding sham-exposed cells (Fig 6F). In all experiments performed (a total of 10 independent experiments), the mean percentage of viable cells in the positive controls measured by the Countess™ automated cell counter amount to 73.83 ± 10.54%.

## Discussion

The COVID-19 pandemic has highlighted the critical need for effective environmental surface disinfection to control infections. Previous experimental studies evaluating various HOCl-based disinfectants have focused primarily on their antimicrobial properties. These studies used HOCl preparations as directly applied solutions to determine the maximum antimicrobial effect on various surfaces or wounds [8,25]. For example, Saxena et al. [26] reported that a 200 ppm HOCl solution exhibited over 99% antimicrobial activity against selected strains of bacteria on hard surfaces after 10 minutes of contact. Other studies investigating similar phenomena typically used HOCl-based disinfectants at concentrations ranging from 50 to 300 ppm [25,27,28]. Benedusi et al. [29] reported that the World Health Organization (WHO) had recognized HOCl as a highly effective disinfectant against many human pathogens at concentrations ranging from 180 to 460 ppm.

However, previous studies have used HOCl at even higher concentrations than those described above. In their study, Herruzo et al. [30] evaluated the microbicidal efficacy of HOCl on bacteria and yeast cultures inoculated on organic agar media at four concentrations: 100 and 300 ppm, but also 500 and 1500 ppm for 1, 5 and 10 minutes. They verified the efficacy of 500 and 1500 ppm HOCl concentrations against biofilms and found that the 1500 ppm HOCl solution was more effective than the more diluted solutions in the first minute. However, after 5 minutes, its efficacy was comparable to the 500 ppm solution. It is worth noting that the authors did not perform their own HOCl cytotoxicity tests on fibroblasts within the concentration range used. Instead, the researchers cited the findings of Day et al. [31], who found 300 or 500 ppm HOCl to be a safe, non-cytotoxic, and effective antiseptic for wounds and mucous membranes over 5–10 minutes. This study also noted the lack of toxicity data for fibroblasts treated with HOCl at a concentration of 1500 ppm.

Since the COVID-19 outbreak, many studies have evaluated the germicidal activity of HOCl-based disinfectants at very high concentrations; however, there are limited data examining the cytotoxic effect of HOCl used as a disinfectant [29,32–35]. In addition, to achieve maximum germicidal effect on a large area in a shorter time, some researchers have developed dry fogging systems that disperse fine droplets of a disinfectant that penetrate inaccessible areas and cover virtually any surface [29,33,35,36]. On the other hand, one of the hazards of dry fogging is the risk of inadvertent exposure of the fogger operator to very small droplets of HOCl (Sauter mean diameter range 10–50 μm) if personal protective equipment is damaged or used incorrectly [37,38]. This physical form of the disinfectant can facilitate its penetration into human tissues, and exposure can cause adverse health effects such as acute skin allergy or inflammation, shortness of breath, coughing, or burning or watery eyes, among others [38,39]. Tibbits et al. [40] demonstrated that approximately 75% of the amount of HOCl applied locally to an epidermal explant was detected up to 300 μm below the surface of the explant 12 hours after application.

Based on the research results presented above, this study aimed to evaluate both the potential cytotoxicity and genotoxicity of HOCl on cultured human skin fibroblasts after fog disinfection. Several chlorine-containing compounds exhibit a broad spectrum of antimicrobial activity and are effective at various concentrations against multiple pathogens, even viruses such as Ebola [41] or SARS-CoV-2 [42]. Human exposure to 500 ppm chlorine spray for handwashing was widespread in Africa during the Ebola epidemic. However, the frequency of single versus multiple chlorine spray exposure significantly increased the risk of skin irritation. Therefore, we also decided to experimentally expose fibroblasts to HOCl dry mist in single versus multiple exposures. We chose single versus multiple fogging to capture the timing of possible changes in cells exposed to HOCl, which is considered a safe antimicrobial chemical factor, especially since there is little data on its effects on human cells *in vitro*. We chose cell exposure at 300 ppm as a potentially safe concentration because it has been shown that no skin irritation was found at 250 ppm for aqueous HOCl solution [42].

A HOCl concentration of 500 ppm was also chosen for fogging. The antimicrobial test results of several commercially available HOCl disinfectants show that this concentration of HOCl is highly effective in inactivating microorganisms [43,44]. In addition, Boecker and colleagues [36] reported in their study that when they aerosolized HOCl solution at a concentration of 500 ppm, they achieved a 3-log reduction in the number of Gram-negative bacteria within only 10 minutes. This was one of the reasons why the exposure time for 500 ppm HOCl aerosolization in our study was set at 9 minutes. The HOCl concentrations that resulted in significant bacterial inactivation were below the EU Occupational Exposure Limit for airborne free chlorine (0.21 ppm) in occupied spaces. However, it is essential to note that this limit is defined for long-term exposure.

In the present study, when the HOCl solution at a concentration of 500 ppm was aerosolized into the experimental chamber, the estimated concentration of free chlorine in the air was below the EU short-term occupational exposure limit (0.5 ppm) within 9 minutes of exposure [45]. However, significant changes in fibroblast morphology were observed under the inverted microscope after exposure to HOCl aerosol at 500 ppm for either one or four cycles. In addition, cell death occurred due to disruption of membrane continuity, regardless of the number of exposure repetitions, as recorded under a scanning confocal microscope. Furthermore, our flow cytometry analysis showed that after four exposure cycles, the percentage of cells with intact plasma membranes decreased to 49.1% in the exposed samples compared to the sham samples. Benedusi et al. [29] conducted a study to evaluate the bactericidal and virucidal efficacy of nebulized HOCl and tested its safety in human keratinocyte skin models. The study concluded that HOCl at a concentration of 300 ppm did not affect cell viability or morphology. This finding has been replicated in our research.

However, Benedusi et al. [29] reported that significant changes in cell structures were observed when cells were exposed to HOCl concentrations of 1000, 2000, 5000, and 10000 ppm. Unfortunately, they did not experiment with the 500 ppm HOCl concentration. Conversely, Tsai et al. showed that in experiments performed according to ISO 10993–5 recommendations, no cytotoxic effects were found at a concentration of 500 ppm HOCl in mouse fibroblasts [34]. However, a concentration-dependent decrease in cell viability was observed. Bilvinaite et al. [46] performed an evaluation of the cytotoxicity of Sterilox^®^ containing HOCl at 144 ppm concentration on human gingival fibroblasts. The study showed significant cytotoxic effects at concentrations above 72 ppm, but only after 24 and 48 hours (p < 0.05).

While some previous studies have warned about the potential health risks and tissue damage resulting from exposure to HOCl fog (such as DNA damage, lipid peroxidation, and protein oxidation) [37], no research has been conducted on aerosolized HOCl-based disinfectants at high concentrations and their genotoxicity in human fibroblasts. In this study, we investigated the potential genotoxic effects of exposure of human fibroblasts to HOCl aerosolization. It is well documented that endogenous HOCl generated by myeloperoxidase is a highly reactive oxidant that can cause damage not only to pathogens but also to host tissues, leading to DNA damage and mutagenesis [36,47–49]. The general mechanism of DNA damage in mammalian cells under the influence of HOCl includes extensive oxidative damage to pyrimidines, cytosine and thymine, and cytosine chlorination, as well as minor oxidative damage to purines [50]. Whiteman et al. [51] showed that the effect of HOCl used at high concentrations on the DNA of mammalian cells leads to the production of 5-chlorouracil, which they considered to be one of the specific markers of DNA damage caused by HOCl. Other authors have confirmed this in their studies [52]. Externally supplied HOCl has to penetrate through several barriers, such as cell membrane, dense cytoplasm, nuclear membrane, and histone wrapping, before reaching the DNA strands. Therefore, DNA is well protected from the HOCl damage. However, as our results show, it had occurred upon the fibroblast exposure to high (500 ppm) HOCl concentration. Further studies on the fibroblast damage should include the above targets as well.

In the current study, four exposures to 500 ppm HOCl aerosol for 9 minutes resulted in significant DNA damage in human fibroblasts. In the previous studies by Zavodnik et al. [47], DNA integrity testing using the alkaline comet assay showed a statistically significant increase in DNA damage in Chinese hamster fibroblasts after exposure to HOCl solution at low concentrations (1 mM for 1 hour). Spencer et al. [53] demonstrated the occurrence of DNA double-strand breaks in human respiratory epithelial cells exposed to a 1 mM HOCl solution for 10 minutes. They suggested that HOCl could diffuse into the nucleus and attack DNA. In our view, the observed genotoxicity towards fibroblasts following exposure to the fog generated using a 500 ppm HOCl solution is not a false positive. According to Azqueta et al. [54], the most commonly accepted threshold at which comet test results could be affected by concurrent cell death is 50% cell viability. In our experiments, fogging cells with an initial HOCl concentration of 500 ppm (9.531 mM) resulted in an average percentage of live cells relative to the parallel negative control (% of Sham) of 91.52 ± 8.42% after one fogging cycle and 55.18 ± 4.50% after four cycles. These results were determined using Trypan blue staining and analysis with a Countess™ Automated Cell Counter (Life Technologies). The findings from flow cytometry corroborated these observations, as illustrated in Fig 4D.

Rasmussen et al. [12] reported that there were no acute histopathological changes in lung or eye cells in rats exposed to micro aerosolized HOCl containing 176 ppm free available chlorine at a rate of 2 mg/L of air flowing at 218 /min. However, the effect of HOCl aerosol exposure on human fibroblasts was not tested. Boecker et al. [36] stated that aerosolized HOCl concentrations below 0.21 ppm (0.5 mg/m^3^) are considered L harmless to humans, with no tissue irritation expected. At the same time, it is important to note that the World Health Organization advises against spraying people with disinfectants in tunnels, cabinets or chambers under any circumstances [55]. The Centers for Disease Control and Prevention (CDC) also does not recommend using these disinfectants to decontaminate common areas for COVID-19. If used, they should be used without human exposure [56].

The main strength of this study is conducting HOCl aerosol experiments in human cells *in vitro*, particularly given the increased use of HOCl aerosol without sufficient research on its human health effects during and after the SARS Cov-2 pandemic. However, a limitation of our work is that only one genotoxicity test was used, which was due to constraints on the number of exposed samples caused by limitations in the experimental chamber and fogging conditions.

## Conclusions

The study showed that four exposures of human fibroblasts to aerosolized HOCl at 500 ppm concentration for 9 minutes caused significant cytotoxicity and genotoxic effects. In contrast, exposure of cells to aerosolized HOCl at a concentration of 300 ppm for 3 minutes did not cause similar damage. Therefore, further research on human fibroblasts and other human cells is essential to determine the precise HOCl concentrations and exposure times required for safe use as a fogging disinfectant. In addition, to broaden our analysis, further studies should be conducted to assess long-term effects that better mimic real-world disinfection scenarios. In addition, *in vivo* studies are essential for a more complete understanding of the effects of HOCl on human tissues. This could include animal models or carefully controlled human exposure studies of the *in vivo* response to HOCl aerosol.

## Supporting information

S1 Fig Morphology of healthy adherent human fibroblasts in control samples under an inverted diagnostic microscope. A. Fibroblasts grown for three days for experiments in which cells were exposed to 300 ppm HOCl for 3 minutes; B. Fibroblasts grown for three days for experiments in which cells were exposed to 500 ppm for 9 minutes. A scale bar of 100 μm is included.
(TIF)
