# Supplementary figures and images for: The HOCl dry fog–is it safe for human cells?
Source: PLoS One. 2024 May 29;19(5):e0304602. doi: 10.1371/journal.pone.0304602 (PMC11135740; doi:10.1371/journal.pone.0304602)

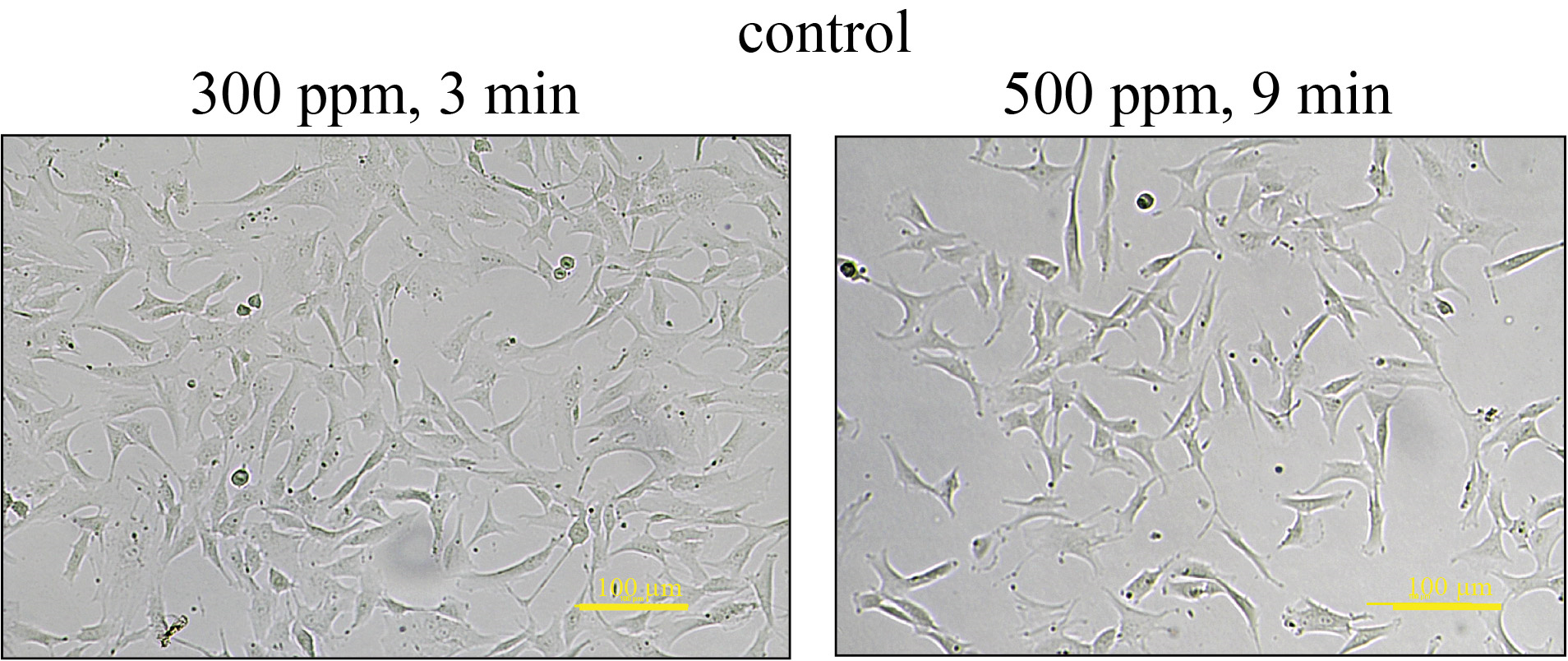

Supplement: S1 Fig — A. Fibroblasts grown for three days for experiments in which cells were exposed to 300 ppm HOCl for 3 minutes; B. Fibroblasts grown for three days for experiments in which cells were exposed to 500 ppm for 9 minutes. A scale bar of 100 μm is included. (TIF) [file pone.0304602.s001.tif]
